# Therapeutic Potential of Natural Xanthones Against Prostate Adenocarcinoma: A Comprehensive Review of Research Trends During the Last Ten Years (2014–2024)

**DOI:** 10.3390/ph18081197

**Published:** 2025-08-14

**Authors:** Gaétan Tchangou Tabakam, Emmanuel Mfotie Njoya, Chika Ifeanyi Chukwuma, Samson Sitheni Mashele, Maurice Ducret Awouafack, Tshepiso Jan Makhafola

**Affiliations:** 1 Centre for Quality of Health and Living, Faculty of Health and Environmental Sciences, Central University of Technology, Bloemfontein 9300, South Africa; tgaetan@cut.ac.za (G.T.T.); enjoya@cut.ac.za (E.M.N.); cchukwuma@cut.ac.za (C.I.C.); smashele@cut.ac.za (S.S.M.); 2 Natural Products Chemistry Research Unit, Department of Chemistry, Faculty of Science, University of Dschang, Dschang P.O. Box 67, Cameroon

**Keywords:** xanthones, garcibractatin A, gaudichaudione H, cantleyanone A, prostatic adenocarcinoma

## Abstract

Prostate cancer is the most common cancer diagnosed in men worldwide and is ranked as the fifth leading cause of cancer-related death in men globally. **Background/Objectives**: We aimed to identify the effectiveness of cytotoxic plant-derived xanthones against prostate cancer over the past ten years. **Methods**: Searches were performed in Google Scholar, Web of Science, Scopus and PubMed/Medline for ten years up to December 2024 using pre-defined inclusion and exclusion criteria. The published articles were assessed in accordance with the PRISMA 2020 procedure. **Results**: From a total number of *n* = 11,932 results, 9 were retained as included studies, which included 51 xanthones. **Conclusions**: Garcibractatin A and bracteaxanthone VII exhibited significant cytotoxic effects on human prostate cancer (PC-3 cells) [IC_50_ value of 2.93 and 4.8 μM] and the human normal prostatic stromal myofibroblast cell line (WPMY-1 cells) [IC_50_ value of 0.76 and 3.2 μM], which were more potent than the reference etoposide [(IC_50_ value of 10.07 μM) and (IC_50_ value of 12.98 μM)]. Parvifolixanthone A showed significant activity on PC-3 (IC_50_ of 4.65 μM), which was more potent than the reference 5-fluorouracil (IC_50_ of 30.59 μM); gaudichaudione H, cantleyanone A, isobractatin, isoforbesione, and neobractatin had strong cytotoxicity (IC_50_ values between 2.10 and 3.39 μM) as compared to etoposide (IC_50_ of 10.07 μM). Despite these positive outlooks, there are still several restrictions, most notably the absence of in vivo evidence in many studies and well-defined mechanisms of action for all the promising bioactive xanthones identified in this work as well as the absence of studies of their cytotoxicity on certain normal cells.

## 1. Introduction

According to the National Institute of Cancer (NIC), 2022, prostate cancer is a disease that develops from initially normal prostate cells, which transform and multiply in an uncontrolled manner until they form a mass called a malignant tumor. The prostate gland is a male reproductive organ located below the bladder and surrounding the urethra. The main function of the prostate is to contribute to the secretion of semen. It forms and ejaculates semen and maintains sperm viability [1]. Prostate cells are often a source of tumors, most often in the middle or the end of human life [2]. Millions of men are affected by prostate cancer each year. In high-income regions, the disease is among the most common solid malignancies, and prognosis varies widely with age, ethnicity, genetic background and stage of progression [3].

The recent work of James et al., 2024 [4] demonstrated that prostate cancer makes up 15% of all cancers and is the most frequent malignancy in men in at least 112 countries or nations. Based on information about global demographic shifts and increasing life expectancy, an estimate puts prostate cancer cases in 2040 at 2.9 million new cases annually, up from 1.4 million four years before (2020). The same authors [4] predicted that a close to 3% global decline in prostate cancer incidence rates from 2020 to 2040 would be required for the case numbers in 2040 to remain the same as those in 2020. Correspondingly, they estimated that prostate cancer deaths will then rise by 85%, from 375,000 in 2020 to close to 700,000 by 2040.

Research on prostate cancer is a very busy field of interdisciplinary study that now includes laboratory and clinical science in addition to computational biology. For many men with prostate cancer, living with the disease involves managing a tailored treatment plan for slow-growing and often indolent tumors, but for many others, disease relapse is expected, following a defined treatment, which may be rapid, aggressive and, in rare cases, unresponsive to standard care. Millions of men are affected by prostate cancer each year. In high-income regions, the disease is among the most common solid malignancies, and prognosis varies widely with age, ethnicity, genetic background and stage of progression [3,5].

Despite a number of advancements in prostate cancer diagnosis and treatment, the disease continues to spread and cause an increasing number of fatalities each year. This necessitates further study on the illness in order to improve diagnosis and treatment strategies and increase patient survival. It is well known that throughout history, humans have exploited nature to meet their basic needs. The use of natural substances as medicines for a variety of illnesses, including cancer in general and prostate cancer (PC) in particular, is also observed [6]. It is well known around the world, as well as among researchers, that natural plants include a variety of chemical functional groups. This is particularly the case with xanthone, which has good or positive biological effects on prostate cancer (PC).

There are different treatment options for prostate cancer (PC). Treatment usually includes surgery and radiation therapy for prostate cancer. Other options include chemotherapy and hormone therapy for prostate cancer, both of which aim to kill cancer cells. Nevertheless, some recognized health organizations have approved many recent treatments for PC, for example, Orgovyx (relugolix), which is a gonadotropin-releasing hormone (GnRH) receptor antagonist indicated for the treatment of advanced prostate cancer and the first oral treatment option for prostate cancer available to men with advanced disease [7]; Erleada (apalutamide), which is indicated to treat men with non-metastatic castration-resistant prostate cancer (NMRDC) who no longer respond to testosterone-lowering medical or surgical hormone therapy; and Nubeqa (darolutamide), which is a medicine used to treat men with non-metastatic castration-resistant prostate cancer [8]. Several research works have demonstrated that many plants around the world contain several classes of compounds, including xanthones, which exhibit good biological activities against different types of cancer cells. It is well known that there are many factors, among which is the resistance of some prostate cancer cell lines to current treatment methods and available drugs, making the evolution of treatment for this disease difficult. Since ancient times, people have always taken advantage of nature in order to meet their primary needs. This also applies to the usage of natural products as medication for a wide range of diseases encompassing cancer [6].

**Figure 1:**
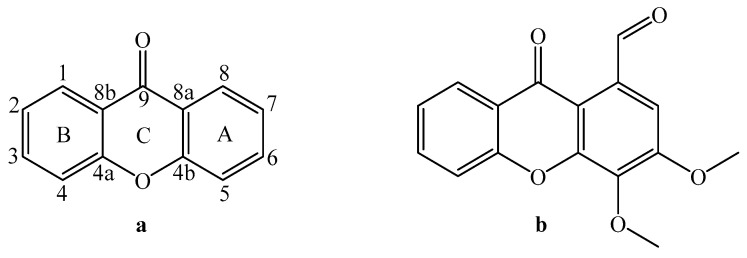
(**a**) Basic skeleton of xanthones. (**b**) Chemical structure of 1-carbaldehyde-3,4-dimethoxyxanthone.

Numerous mechanisms have been suggested that may owe to xanthones’ anticancer activity. Various xanthones, mainly α-mangostin and gartanin, have been reported to incur cell cycle arrest at the G1 phase and decrease cyclin D1 activity in prostate cancer [9,10,11,12,13]. Hung et al. reported that α-mangostin had an anti-metastatic effect on PC-3 cells by decreasing matrix metalloproteinases [14]. Tsai et al. showed that mangosteen pericarp extract could slow the progression of prostatic hyperplasia in vivo [15]. The Johnson group has reported that α-mangostin and gartanin decrease the viability of 22Rν1 and LNCaP cells, promote apoptosis, modulate endoplasmic reticulum stress, inhibit CDKs, and show antitumor effects in prostate cancer mouse models [16,17,18,19]. The xanthone family is a heterocyclic class of secondary metabolites found mainly in lichens, mushrooms, and higher plant groups. They are formed from dibenzo-γ-pyrone, which is the γ-pyrone condensed with two benzene rings [20] (Figure 1a). There are several types of xanthone, according to the level of their oxygenation or the quality of the ring residue. The structural diversity of this class of compound has enabled it to exhibit considerable biological properties; there is the case of 1-carbaldehyde-3,4-dimethoxyxanthone (Figure 1b), which has recently emerged as a powerful inhibitor of the growth of androgen-sensitive (LNCaP) and androgen-independent (PC-3) tumor cells in prostate cancer [21].

The main objective of this work is to identify the new and known plant-derived bioactive xanthones and their derivatives during the last ten years, which can be the subject of additional analyses (missing biological tests, hemisynthesis of promising compounds, …) to be used in the treatment of prostate cancer to improve the shortcomings of current treatment.

## 2. Methods

### 2.1. Eligibility Criteria

The eligibility criteria for our work were well defined according to the requirements of systematic reviews, and, therefore, studies were conducted on each geographical area of the world. Only compounds from the xanthone family and their derivatives that exhibit activities ranging from weak to significant on prostate cancer cell lines were specifically listed in the present study. Studies highlighting activities on prostate cancer cell lines exhibited by classes of compounds other than xanthones were not considered. Results published in the form of reviews, letters, editorials, conference abstracts, anonymous reports, unpublished works, commentaries, and criticisms have not been considered. All experimental studies in vitro/in vivo or in silico that evaluated the effects of natural plant-derived xanthone and their derivatives on prostatic adenocarcinoma or prostate cancer as a primary or secondary objective were deemed eligible for our survey.

### 2.2. Information Sources and Searches

The Preferred Reporting Items for Systematic Reviews and Meta-Analyses (PRISMA) statement, PRISMA for abstract [22], and PRISMA for searching [23] were used to establish and carry out this present research work. Three electronic databases, including Web of Science, Medline/PubMed, and Google Scholar, were exploited in the context of our literature review. From the above-mentioned databases, all the scientific work published between 2014 and 2024 related to our subject was brought together, with no restrictions in terms of publication date or language used. The methods applied in this article were validated by all the authors listed. The search strategies and terms used for this work included the following: “bioactive xanthone” OR “Natural bioactive xanthones” OR “Bioactive plant-derived xanthones” OR “Isolated bioactive xanthones” AND “Prostate cancer” OR “Prostatic adenocarcinoma” OR “Caucasian prostatic adenocarcinoma” OR “glandular cancer” OR “glandular carcinoma”.

### 2.3. Major Selection or Study Choices

**Figure 2:**
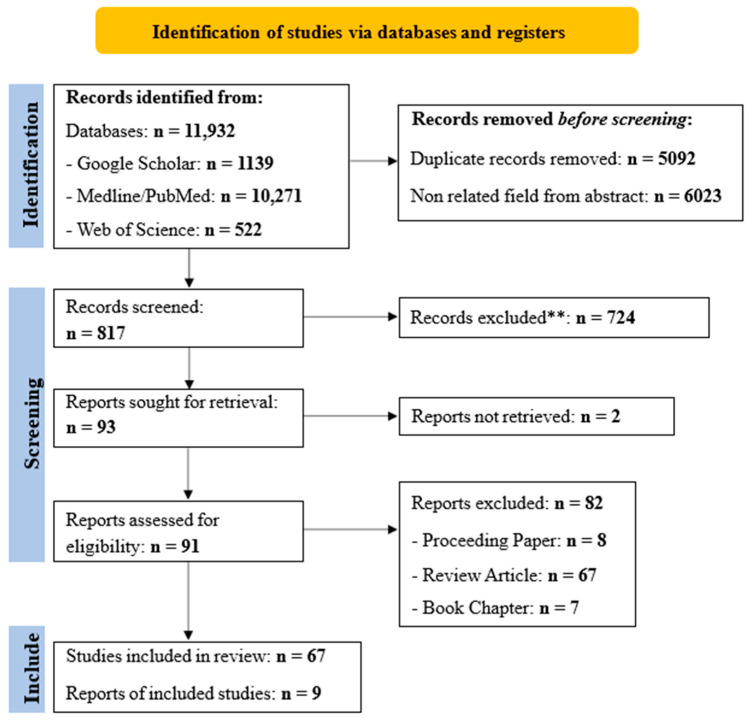
Schematic flow diagram for the selection of this study according to PRISMA checklist 2020. **: means the number of research excluded. Registration code Prospero ID 1091345.

According to the procedure by Page et al. (2021) [22], the identified works were transferred to EndNote, articles that appeared as duplicates were eliminated, and summaries and research titles were created. During the second stage of independent selection, published articles containing information related to the data sought in our work that met the eligibility criteria were carefully utilized in their entirety. In the third and final stage, the authors of this work took the time to meticulously verify the information derived from each individual selection to determine the final list of studies that would be incorporated into the study. The steps developed in the PRISMA flow diagram [6,22] were respected and followed so that unnecessary documents for the present review were gradually eliminated until the total number of articles was obtained (Figure 2).

### 2.4. Data Collection and Methodological Quality Assessment

The number of each xanthone and derivative, the different classes of xanthone, the plant source (family), the year, the country, the type of cancer cell line, and the references that were used were all extracted in order to gather high-quality data and conduct a thorough evaluation. Data extraction was performed individually by each author. We determined the chemical structures of these xanthones and derivatives using Figure 2 and present the results in a synoptic table (Section 3.1).

## 3. Results

### 3.1. Characteristics of Results from Literature Search

**Figure 3:**
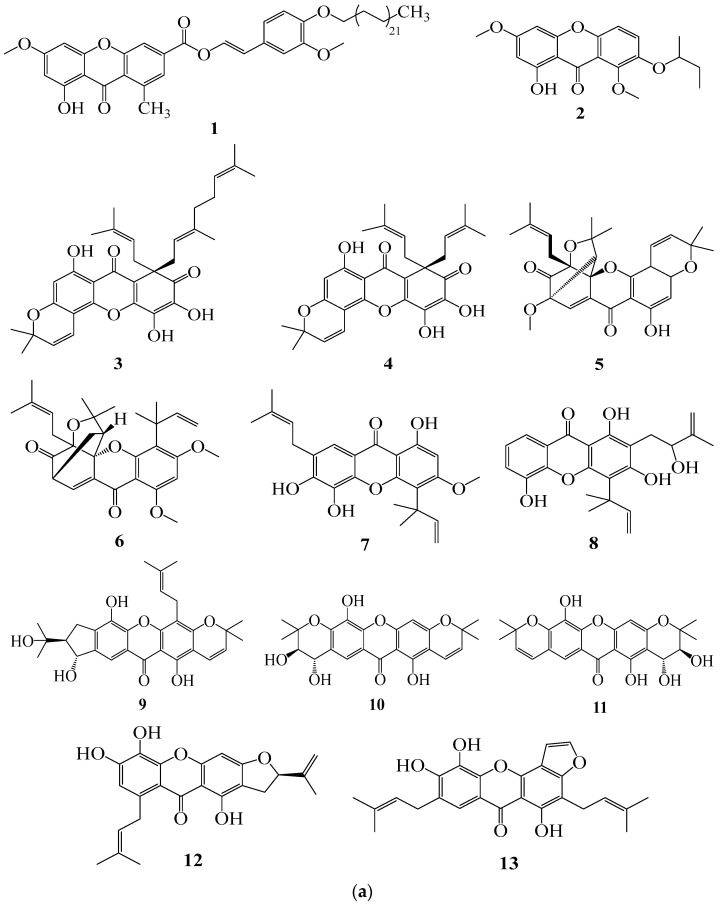

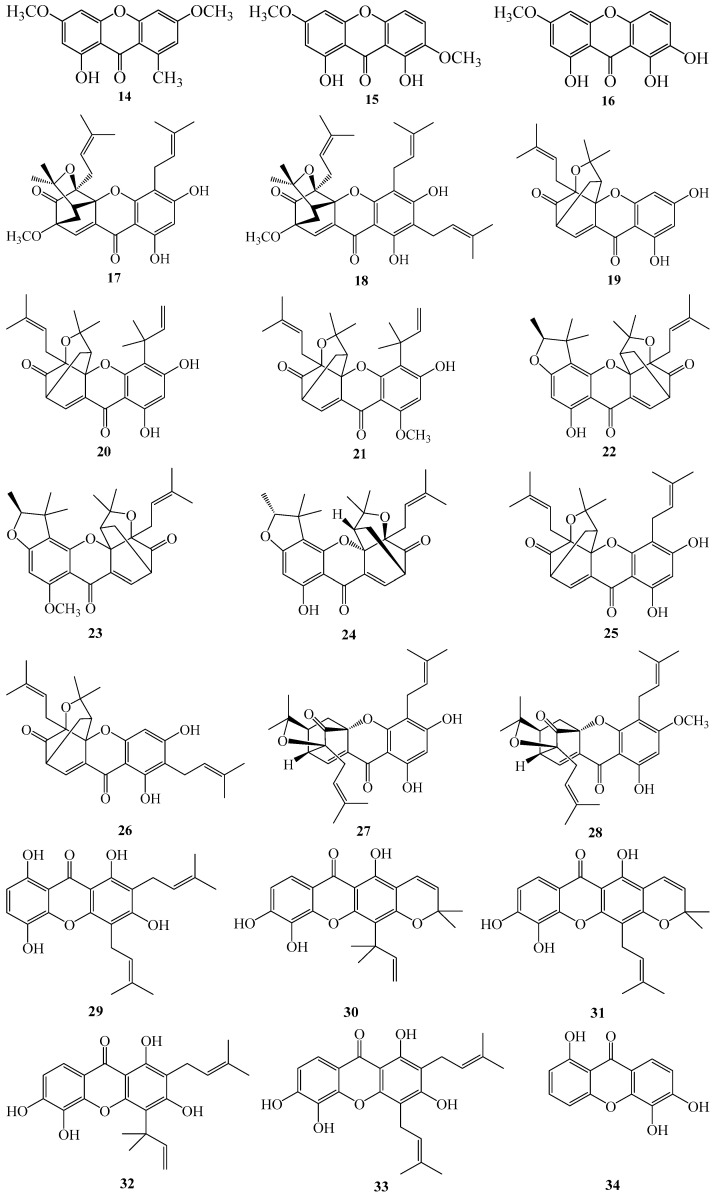

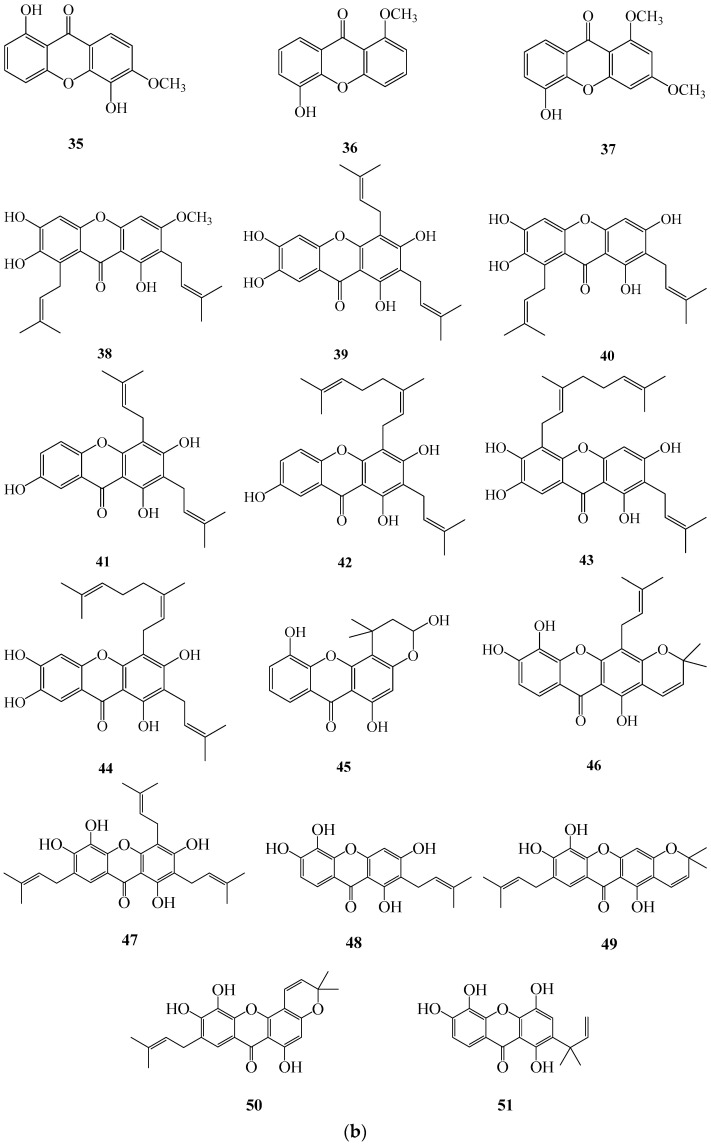
(**a**) Structures of new reported xanthones. (**b**) Structures of known reported xanthones.

Nine (9) results were found through a literature search regarding the activities of xanthones and their derivatives against prostatic adenocarcinoma (Figure 2). Fifty-one (51) secondary metabolites belonging to five subclasses of xanthones are listed as follows: [Simple oxygenated xanthones (**1**–**2**, **14**, **15**–**16**, **34**–**37**); prenylated xanthones (**3**–**4**, **7**–**9**, **12**–**13**, **29**–**33**, **38**–**44**, **46**–**51**), caged prenylated xanthones (**5**–**6**, **17**–**26**); neo caged-prenylated xanthones (**27**–**28**), and pyranoxanthones (**10**–**11**, **45**)] were revealed by our studies enumerated below: [medicaxanthone (**1**) and lichenxanthone (**14**)] [24], [coxanthone B (**2**); 1,7,8-Trihydroxy-3-methoxyxanthone (**16**)] [25], [oliganthin H (**3**), oliganthin I (**4**), oliganthone B (**5**), gaudichaudione H (**17**) and cantleyanone A (**18**)] [26], swertiperenine (**15**) [25,27], [garcibractatin A (**6**), cochinchinoxanthone (**19**), bractatin (**20**), 1-*O*-methylbractatin (**21**), isobractatin (**22**), 1-*O*-methylisobractatin (**23**), epiisobractatin (**24**), forbesione (**25**), isoforbesione (**26**), neobractatin (**27**), 3-*O*-methyl-neobractatin (**28**)] [28], [bracteaxanthone VII (**7**), bracteaxanthone VIII (**8**), gartanin (**29**), 3-Hydroxyblanco-xanthone (**30**), xanthone V1 (**31**), gerontoxanthone I (**32**), xanthone V1a (**33**)] [29]; [1,5,6-trihydroxyxanthone (**34**); 1,5-dihydroxy-6-methoxyxanthone (**35**), 5-hydroxy-1-methoxyxanthone (**36**), 5-hydroxy-1,3-dimethoxyxanthone (**37**)] [30], [dulcisxanthone B (**38**), cudratricusxanthone E (**39**), γ-mangostin (**40**); 1,3,7-trihydroxy-2,4-diisoprenylxanthone (**41**), cochinchinone A (**42**), cochinchinone B (**43**), pruniflorone Q (**44**), pruniflorone N (**45**), xanthone V1 (**46**)] [31], [paucinervin L (**9**), (+) Paucinervin N (**10**), (–) Paucinervin N (**11**), paucinervin O (**12**), paucinervin P (**13**), parvifolixanthone A (**47**), 2-prenyl-1,3,5,6-tetrahydroxylxanthone (**48**), 7-prenyljacareubin (**49**), paucinervin I (**50**)] [32], Subelliptenones F (**51**) [33] (Table 1, Figure 3a,b). All studies have documented in vitro activity, and the research presented in this work was carried out in the African and Asian continents, particularly in Cameroon, India, China, Japan and Thailand.

Xanthones are one of the biggest classes of compounds in natural product chemistry. A number of xanthones have been isolated from natural sources of higher plants, fungi, ferns, and lichens. They have gradually risen to great importance because of their medicinal properties [34]. This review allowed us to identify 51 new and known xanthones belonging to five different subclasses with anti-prostate cancer activity during the last ten years. The activities of the different xanthones included in this work were classified according to the cut-off point below: for anticancer activity of plant metabolites, significant or strong cytotoxicity: IC_50_ < 4 μg/mL (or IC_50_ < 10 μM); moderate cytotoxicity: 4 μg/mL < IC_50_ < 20 μg/mL (or 10 μM < IC_50_ < 50 μM); low cytotoxicity: 20 μg/mL < IC_50_ < 100 μg/mL (or 50 μM < IC_50_ < 250 μM) [35].

### 3.2. Simple Oxygenated Xanthones

Simple oxygenated xanthones are xanthones subdivided according to the degree of oxygenation into non-, mono-, di-, tri-, tetra-, penta-, and hexa-oxygenated substances [36,37,38]. In these subclasses of xanthones, the substituents are simple hydroxyl, methoxyl, or methyl groups. In our present work, we report nine simple oxygenated xanthones. Medicaxanthone or 3-tetracosyloxyferulate of 1,6-dihydroxy 3-methoxy-8-methylxanthone (**1**) and coxanthone B or 2*S*-(*sec*-butoxy)-8-hydroxy-1, 6-dimethoxy-9*H*-xanthen-9-one (**2**) were newly isolated from *Citrus medica* (Rutaceae) and exhibited weak in vitro cytotoxic activity against Caucasian human prostate cancer cell line PC-3, with IC_50_ values of 65.0 and 48.0 µM, respectively [24,25]. Lichenxanthone (**14**) was first isolated from the genus *Diploschistes* s. lat. [39] and isolated again from *Citrus medica* (Rutaceae) and showed weak in vitro cytotoxicity on PC-3 with an IC_50_ value of 70.2 µM [24]. Swertiperenine (**15**) and 1,7,8-trihydroxy-3-methoxyxanthone (**16**) were isolated from *Codonopsis ovata* (Campanulaceae) and showed weak in vitro cytotoxicity activity against PC-3 with IC_50_ values of 48.0 and 64.0 µM, respectively. Swertiperenine (**15**) was previously identified from the same plant species in 2014 by Dar et al. [27], while 1,7,8-trihydroxy-3-methoxyxanthone (**16**) was initially isolated from *Chironia krebsii* (Gentianaceae) [40]. 1,5,6-Trihydroxyxanthone (**34**), 1,5-dihydroxy-6-methoxyxanthone (**35**), 5-hydroxy-1-methoxyxanthone (**36**), 5-hydroxy-1,3-dimethoxyxanthone (**37**) were isolated from *Mesua ferrea* (Guttiferae) and exhibited significant, moderate and weak in vitro cytotoxic activity against PC-3 with IC_50_ values of 5.94, 96.08, 26.81 and 60.89 µM, respectively, as compared to the standard doxorubicine with an IC_50_ value of 0.9 µM [30]. Compounds **34**, **35**, **36** and **37** were first reported from *Musea ferrea* (Guttiferea) [41], *Tovomita excelsa* (Guttiferae) [42], *Mammea siamensis* (Guttiferae) [43]. Except for compound **34,** which exhibited strong in vitro cytotoxicity against PC-3, all remaining eight simple oxygenated xanthones possessed weak to moderate activities against PC-3. However, none of these studies evaluated the in vivo activities of simple oxygenated xanthones enumerated herein.

### 3.3. Prenylated Xanthones

Prenylated xanthones are secondary metabolites having one or many prenylated groups on their xanthone base; they are particularly common in plants belonging to the Clusiaceae family [44]. Twenty-five bioactive prenylated xanthones previously identified were summarized in this work. Oliganthins H (**3**) and I (**4**) were isolated for the first time from *Garcinia oligantha* (Clusiaceae) and had significant in vitro cytotoxic activities on human prostate cancer PC-3, with IC_50_ values of 5.9 and 3.2 µM, respectively, as compared to paclitaxel with an IC_50_ of 0.03 µM [26]. From the other species of the same genus *Garcinia*: *G. bracteata* (Clusiaceae), three compounds: bracteaxanthones VII (**7**) and VIII (**8**) and paucinervin L (**9**) were isolated as new compounds and were reported to exhibit significant (IC_50_ = 4.8 and 9.2 µM) and moderate (IC_50_ = 30.06 µM) in vitro cytotoxic activity against human prostate cancer PC-3, respectively. Etoposide (IC_50_ = 4.4 µM) and 5-flucouracil (IC_50_ = 30.59 µM) were used as references for compounds 7 and 8 as well as for compound **9**, respectively [31,32]. Otherwise, compound **7** also showed significant in vitro cytotoxic activity against the non-cancerous human prostatic stromal myofibroblast cell line (WPMY-1) with an IC_50_ value of 3.2 µM, as compared to etoposide (IC_50_ = 3.8 µM) [32]. From the same plant species, *G. bracteata* (Clusiaceae), a known analogue of prenylated xanthones, and its in vitro cytotoxic activities were demonstrated against two human [prostate cancer (PC-3) and non-cancerous prostatic stromal myofibroblast (WPMY-1)] cell lines: Gartanin (**29**) [moderate and significant activities against PC-3 and WPMY-1 (IC_50_ = 12.0 and 6.5 µM)], 3-hydroxyblancoxanthone (**30**) [significant against both cells (IC_50_ 9.7 and 6.5 µM)], Xanthone V1 (**31**) [significant against both cells (IC_50_ = 6.4 and 6.0 µM)], Gerontoxanthone I (**32**) [significant against both cells (IC_50_ = 6.2 and 5.7 µM)] and Xanthone V1a (**33**) [moderated against both cells (IC_50_ = 13.7 and 11.9 µM)], as compared to (IC_50_ = 4.4 and 3.8 µM, respectively) [28]. From the plant species *G. paucinervis* (Clusiaceae), paucinervin O (**12**) and paucinervin P (**13**) were isolated as new compounds and were reported to exhibit moderate in vitro cytotoxic activities with IC_50_ values of 16.63 and 12.23 µM, respectively, as compared to 5-flucouracil (IC_50_ = 30.59 µM) [32]. Compounds **30** and **32** were isolated for the first time from the roots of *Calophyllum blancoi* (Guttiferae) [45] and from *Cudrania cochinchinensis* (Moraceae) [46], respectively, while compounds **31** and **33** were isolated for the first time from *Vismia guineensis* (Clusiaceae) [47]. Isolated for the first time from very ripe fruits of *G. mangostana* Linn [48], eight known prenylated xanthones (Dulcisxanthone B (**38**); cudratricusxanthone E (**39**); γ-Mangostin (**40**); 1,3,7-trihydroxy-2,4-diisoprenylxanthone (**41**); Cochinchinone A (**42**); Cochinchinone B (**43**); Pruniflorone Q (**44**) and xanthone V1 (**46**)) were isolated from *Cratoxylum cochinchinense* Blume (Clusiaceae) and reported to exhibit moderate in vitro cytotoxic activities against human prostate cancer PC-3 [IC_50_ = 21.87, 11.77, 27.11, 20.60, 11.95, 14.99, 14.57, and 20.72 µM, respectively], as compared to 5-Flucouracil (IC_50_ = 25.98 µM) [32]. Compounds **38**, **39**, **41**, (**42** and **43**), **44** and **46** were isolated for the first time from the fruit of *G. dulcis* (Guttiferae) [49], *Cudrania tricuspidata* Bureau (Moraceae) [50], Guttiferaceous plants [51], *Cratoxylum cochinchinense* [52], *Cratoxylum cochinchinense* [53], and *Vismia guineensis* (Guttiferae) [47]. Four known compounds were isolated and identified from *G. paucinervis* (Clusiaceae), displaying significant and moderate in vitro cytotoxic activities against human prostate cancer PC-3: parvifolixanthone A (**47**) [significant (IC_50_ = 4.65 µM)], 2-prenyl-1,3,5,6-tetrahydroxylxanthone (**48**) [moderate (IC_50_ = 35.03 µM)], 7-prenyljacareubin (**49**) [significant (IC_50_ = 9.65 µM)], paucinervin I (**50**) [moderate (IC_50_ = 22.15 µM)], with the 5-flucouracil taken as a reference (IC_50_ = 30.59 µM) [32]. Otherwise, one known compound, Subelliptenones F (**51**), isolated from *Garcinia subelliptica* (Clusiaceae) displayed potent inhibition of AR transcriptional activity (tested at 1–10 µM) against human Lymph Node Carcinoma of the Prostate (LNCaP) [33]. Compounds **47**, **48**, **49**, **50** and **51** were isolated as new compounds from the twigs of *Garcinia parvifolia* (Guttiferae) [54], *Hypericum androsaemum* (Hypericaceae) [55], *Rheedia gardneriana* (Guttiferae) [56], *G. paucinervis* (Clusiaceae) [57] and from the root bark of *G. subelliptica* (Clusiaceae) [58]. Among the twenty-five prenylated xanthones, one was studied for its mechanism of action on prostate cancer. Nauman et al. (2021) [59] isolated and identified compound **40** from *G. mangostana*, which demonstrated a direct inhibition of CDK2/CyclinE1 in prostate cancer cells. Fourteen prenylated xanthones exhibited significant in vitro cytotoxic activities, and the in vitro cytotoxicity of the remaining eleven was moderate. None of these compounds have been the subject of an in vivo study.

### 3.4. Caged-Prenylated Xanthones

Caged-prenylated xanthones are “privileged structures” characterized by the presence of the unusual 4-oxotricyclo[4.3.1.0]dec-8-en-2-one scaffold [60,61]. The natural sources of these compounds are confined mainly in the *Garcinia* genus in the family of Guttiferae [62]. Twelve caged-prenylated xanthones have been identified and included in this work. Oliganthone B (**5**) and garcibractatin A (**6**) were isolated as new compounds for the first time from *Garcinia oligantha* (Clusiaceae) and *G. bracteate* (Clusiaceae), respectively; the in vitro cytotoxic activity of **5** was significant, with an IC_50_ value of 4.6 µM against human prostate cancer PC-3 [26], while compound **6** was strongly active against human PC-3 and WPMY-1 with respective IC_50s_ of 2.93 and 0.76 µM, more potent than etoposide (IC_50_ = 10.07 and 2.98 µM) taken as a reference [29]. Known compounds gaudichaudione H (**17**) isolated for the first time from *Garcinia gaudichaudii* (Clusiaceae) [63] and cantleyanone A (**18**) from *G. cantleyana* (Clusiaceae) [64] have been re-isolated again from *G. oligantha* (Clusiaceae) and exhibited significant in vitro cytotoxic potential against PC-3 with IC_50_ values of 2.1 and 2.3 µM, as compared to paclitaxel (IC_50_ = 0.03 µM) [26]. Eight compounds (cochinchinoxanthone (**19**), bractatin (**20**), isobractatin (**22**), 1-*O*-methylisobractatin (**23**), epiisobractatin (**24**), Forbesione (**25**), Isoforbesione (**26**) and 1-*O*-methylbractatin (**21**)) were isolated from *G. bracteate* (Clusiaceae) and exhibited significant activities, with IC_50_ values of [(4.84, 4.24, 3.39, 8.34, 7.85, 4.34 and 2.75 µM) on PC-3 and 6.5 µM on WPMY-1], respectively [19,28]. Compounds **19**, **20**–**23**, **24** and **25**–**26** were isolated for the first time from *Cratoxylum cochinchinense* (Clusiaceae) [65], *G. bracteate* (Clusiaceae) [66], *G. bracteate* (Clusiaceae) [67] and *G. forbesii* (Gutiferae) [68], respectively. The mechanism of action of compound **22** was studied in prostate cancer. In fact, treatment of PC-3 cells with isobractatin (**22**) led to an enhancement in cell apoptosis and arrested the cell cycle in the G0/G1 phase. The G0/G1 phase cycle-related protein analysis showed that the expressions of cyclins D1 and E were reduced by **22**, whereas the protein level of cyclin-dependent kinase (CDK) inhibitor P21 was induced. Additionally, **22** enhanced PC-3 cell apoptosis activating Bax, caspases 3 and 9 and by inhibiting Bcl-2 [69]. See Table 2.

### 3.5. Neo Caged-Prenylated Xanthones

Neo caged-prenylated xanthones are a “privileged structure” close to the caged-prenylated xanthone characterized by the presence of the unusual 2-oxotricyclo[4.3.1.0]dec-8-en-3-one scaffold [61]. Two neo caged-prenylated xanthones were identified in our present work. Compounds neobractatin (**27**) and 3-*O*-methyl-neobractatin (**28**) first isolated from *Garcinia bracteata* (Clusiaceae) [70] were isolated again from the same plant, and their significant in vitro cytotoxic activities against human prostate cancer PC-3 with the respective IC_50_ values of 2.88 and 4.45 µM with etoposide (IC_50_ = 4.45 µM) were taken as a reference [28]. Only in vitro studies have been performed on these two compounds.

### 3.6. Pyranoxanthones

Pyranoxanthones are compounds with an intra-oxygenated ring attached to the basic skeleton of xanthone. They were discovered in nature at the beginning of the 1970s. They have a very limited distribution and have been isolated from certain plants of the family Guttiferae and also from the mycelia of lower fungi [71]. Mainly dihydropyranoxanthones with a single furan ring are encountered in plants of the family Guttiferae [71]; but, in our current work, we identified three anti-prostate-cancer pyranoxanthones. The compounds (+) and (−) paucinervin N (10 and 11) were isolated for the first time from Garcinia paucinervis (Clusiaceae) and exhibited moderate in vitro cytotoxic activity on human PC-3, with IC_50_ values of 10.92 and 38.77 µM, respectively, as compared to 5-fluorouracil (IC_50_ = 30.59 µM). Pruniflorone N (45) was isolated from Cratoxylum cochinchinense (Clusiaceae) and showed moderate in vitro cytotoxic activity with an IC_50_ value of 22.94 µM with 5-fluorouracil (25.98 µM) taken as a reference [31]. It was first isolated from Cratoxylum formosum (Hypericaceae) [72]. All the activities studied regarding these compounds were only in vitro.

In order to enhance the quality of our literature review and streamline the research findings, we created Table 3, below, showing a comprehensive database (molecular formula and molecular weight (cal.)) of all 51 bioactive xanthones and their derivatives mentioned in the document.

## 4. Discussion

### 4.1. Discussion and Structure–Activity Relationships (SARs) of Plant-Derived Bioactive Xanthones Against PC-3 and WPMY-1 During the Past Ten Years

#### 4.1.1. Downregulation of Hormone-Dependent Prostate Cancer by Xanthones

Hormone-dependent prostate cancer is the most common cancer in men. Endogenous and exogeneous steroids as well as proteo- and peptide hormones play essential roles in the development and progression of these hormone-dependent malignancies. Pharmacological manipulations of these endocrine mechanisms are a cornerstone of the treatment of these tumors, which eventually develop resistance to endocrine therapy [73]. Prostatic hyperplasia, characterized by progressive hyperplasia of glandular and stromal tissues, is the most common proliferative abnormality of the prostate in aging men. A high-fat diet is usually a major factor inducing oxidative stress, inflammation, and an abnormal state of the prostate [15]. According to the literature, *Mangosteen pericarp* powder supplementation could be used to attenuate the progression of prostatic hyperplasia; *Mangosteen pericarp* powder has abundant xanthones, which can be antioxidant, anti-inflammatory, and antiproliferative agents [14]. Sarmento-Cabral et al. [74] reported that ki67 expression increased when nude mice were injected with PC-3 cells and fed a high-fat diet (60% of total kcal from fat). *Mangosteen pericarp* powder treatment inhibited proliferating cell nuclear antigen expression. Using a human prostate cancer cell model, Johnson et al. [13] indicated that due to its structure, α-mangostin, the major xanthone of *Mangosteen pericarp* powder, inhibited cyclin/cyclin-dependent kinase 4 (CDK4), and treating mice with α-mangostin (100 mg/kg) via oral gavage significantly decreased the average tumor volume in an in vivo 22Rv1 tumor xenograft model. From the above-mentioned results, *Mangosteen pericarp* powder (which has abundant xanthones) treatment could suppress abnormal cell proliferation in the prostate.

#### 4.1.2. Discussion and Structure–Activity Relationships (SARs)

Prostate cancer is a complex disease that affects millions of men globally, predominantly in high human development index regions [1]. Our current work allowed us to collect all the research from Cameroon, India, China, Japan, and Thailand during the last ten years (2014–2024) referring to bioactive xanthone and its derivatives against prostate cancer. The different prostate cancer cell lines, PC-3, LNCaP and normal prostate cancer cell WPMY-1, are those on which the 51 identified compounds have been tested.

The Clusiaceae family is a rich source of secondary metabolites, in which one of the major classes of compound is found to be xanthones, produced by plants mainly as a defense mechanism [75]. Regarding the results from our present study, 51 bioactive xanthones and their derivatives were identified, 42 belonging to the Clusiaceae family and the remaining 9 to the Calophyllaceae (4), Campanulaceae (3), and Rutaceae (2) families, showing exactly that Clusiaceae family plants are a rich source of bioactive xanthones, with good activity on prostate cancer in general and on PC-3, normal cell WPMY-1 and cancer cell LNCaP in particular.

This work allowed us to identify 41 different plant species from which these bioactive xanthones were isolated. These species belong to five different genera of plants: the genera *Garcinia* (23 species), *Cratoxylum* (9 species), *Mesua* (4 species), *Codonopsis* (3 species), and *Citrus* (2 species). This result suggests that plants belonging to the *Garcinia* genus are an important source of bioactive xanthones against prostate cancer cells (PC-3, normal cell WPMY-1, and LNCaP), which confirms the results from the literature, saying that *Garcinia* species are well known as rich sources of xanthones [76], which are phenolic constituents reported to possess cytotoxic activities [60,64].

The general observations and comparisons of the activities of the different xanthones reported and grouped in this work show the following: caged and non-caged xanthones are the subclass exhibiting the most significant in vitro cytotoxic activities on prostate cancer cells (IC_50_ 0.76–7.85 µM), followed by prenylated xanthones (IC_50_ of 11.77–35.03 µM), pyranoxanthones (IC_50_ 10.92–38.77 µM), and oxygenated xanthones (IC_50_ 26.01–96.08 µM).

**Figure 4:**
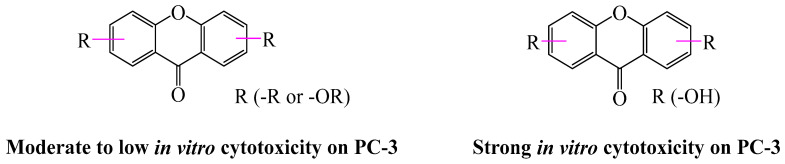
Suggestion of the SAR of oxygenated xanthone on PC-3.

Among the oxygenated xanthones listed, five (**1**, **14**, **16**, **35** and **37**) show very weak activities (IC_50_ of 65.0–96.08 µM); this activity nearly doubles for compounds **2** and **15** (IC_50_ of 48.0 µM); this activity also doubles (IC_50_ of 26.01 µM) for compound **34** and becomes significant (IC_50_ of 5.94 µM). This information allowed to suggest that the alkyl (-R) and/or alkoxy (-OR) groups could not enhance the in vitro cytotoxic activity on PC-3; otherwise, compound **34** with free 1,5,6-trihydroxyl groups (poly-hydroxylation) was found to be more active than the other oxygenated xanthones, showing that the numbers and locations of the hydroxyl groups were key features to exhibit cytotoxicity in this subclass of xanthone on the human prostate cancer cell line (PC-3). This observation fit partly with cell proliferation inhibition activities previously reported [64,77] (Figure 4).

**Figure 5:**
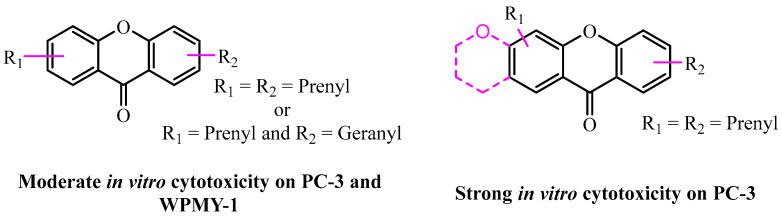
Suggestion of the SARs of prenylated xanthone on PC-3 and WPMY-1.

Regarding the prenylated xanthones, twenty-four compounds showed activities ranging from moderate to strong: compounds **12**, **13**, **29**, **33**, **39**–**44**, **46**, **48** and **50** exhibited moderate activities (IC_50_ of 11.77–35.03 µM); the activity of compounds **9**, **30**–**32**, and **49** remained moderate but twice as active (IC_50_ of 6.20–9.70 µM) compared to those mentioned first, while we observed significant activities with compounds **3**–**4**, **7**–**8** and **47** (IC_50_ of 3.2–5.9 µM). This suggests that the prenylated xanthones exhibited moderate activity on PC-3 and normal cell WPMY-1 when they had at least two prenyl groups or one prenyl and one geranyl group on their basic skeleton. This activity increases when there is an association of a prenyl group and a pyran cycle or both a prenyl and an allyl group on the basic skeleton. We also noticed that their activity increases very significantly when the basic skeleton has at least two prenyl groups and a pyran core. Finally, we can suggest that when the number of isoprenyl groups increases, the cytotoxicity become stronger on PC-3 and normal cell WPMY-1. This observation is in accordance with some previous work confirming the isoprenyl group plays an important role in the cytotoxicity of naturally occurring xanthones [78] (Figure 5).

**Figure 6:**
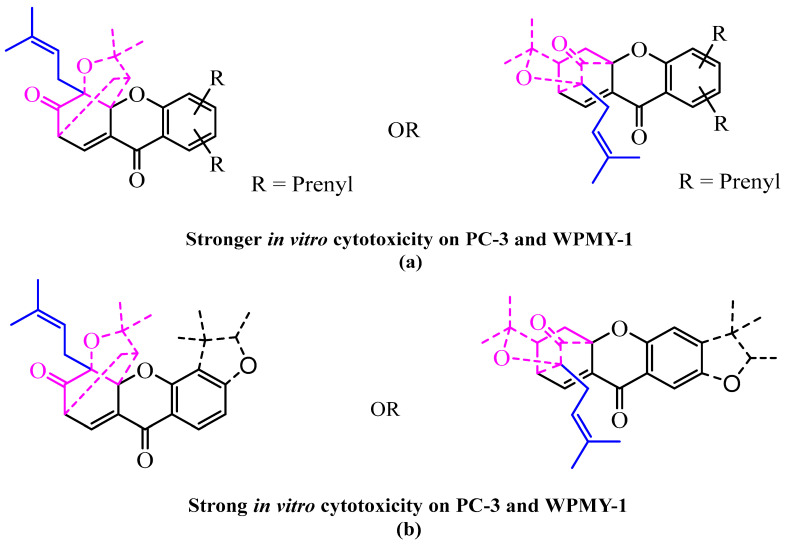
Suggestion of the SAR of caged and non-caged prenylated xanthone on PC-3 and WPMY-1. (**a**) Unusual “4-oxotricyclo[4.3.1.0]dec-8-en-2-one” or “2-oxotricyclo[4.3.1.0]dec-8-en-3-one” scaffold in association with at least two prenyl groups on the xanthonic base skeleton. (**b**) Unusual “4-oxotricyclo[4.3.1.0]dec-8-en-2-one” or “2-oxotricyclo[4.3.1.0]dec-8-en-3-one” scaffold associated with a furanic core on the xanthonic base skeleton.

The fourteen caged and non-caged prenylated xanthones collected and presented in this work showed significant activities (IC_50_ of 0.76–8.34 µM). The most active ones were compounds **6**, (**17**–**18**), **21, 26** and **27** (IC_50_ of 0.76–2.88 µM) compared to compounds **5**, **19**–**20**, **22**–**25**, and **27**–**28** (IC_50_ of 3.39–8.34 µM), suggesting that the presence of the unusual “4-oxotricyclo[4.3.1.0]dec-8-en-2-one” or “2-oxotricyclo[4.3.1.0]dec-8-en-3-one” scaffold in association with at least two prenyl groups on the xanthonic base skeleton increases the activity of the latter (Figure 6a) compared to the presence of this scaffold associated with a furanic core (Figure 6b).

In this context, it is worth mentioning that the cytotoxic potential of compounds **25** and **26** (IC_50_ of 4.34 and 2.75 µM), which possess prenyl groups in the non-caged region (A ring) of the caged xanthone, was more active than the cytotoxic activity of the corresponding caged xanthone **19** (IC_50_ of 4.84 µM), showing that the activity against the human prostate cancer cells was significantly impacted by the varied prenyl substitution in the non-caged region of the caged xanthones.

### 4.2. Limitations

The results collected during this work reveal the toxicities of xanthone and derivatives only under in vitro conditions. The adverse effects of all the compounds identified were not evaluated. These limitations mean that the degree of safety of these compounds is unknown, although they exhibit cytotoxic activities against cancer cells, making it urgent for researchers to undertake more advanced research in order to evaluate these activities on normal cells. Since we do not know the pharmaceutical formulation a medicine can take during its transfer into a living organism (active, inactive, less active, or metabolism into a harmful form), the lack of information or in vivo work carried out with these natural compounds is another key constraint. Researchers should investigate this in the future to obtain comprehensive information (both in vitro and in vivo data) on these compounds that have been examined and seem to be promising, as reported in this work. The other important limitation is the focus on the lack of the mechanism of action on different cancer cell lines of many of the identified bioactive compounds reported in this work (only two among the fifty-one enumerated).

### 4.3. Perspectives

The toxicity profiles of all the identified bioactive xanthones should be evaluated. The semi-synthesis of the caged and neo caged-prenylated xanthones should be fully evaluated. Elucidation of the exact biological mechanisms and the associated targets of xanthones will yield better opportunities for these compounds to be developed as potential anticancer drugs. Further clinical studies with conclusive results are required to implement xanthones as treatment modalities in cancer. In the near future, researchers should aim to enhance the cytotoxicity of xanthone on cancer cell lines and to reduce the toxicity on normal cells. Different compounds should be tested on other prostate cancer cell lines such as DU-145 as well as the xenograft model of the 22Rv1 cell line.

## 5. Conclusions

The present work permits us to report plant-derived xanthones and their derivatives with cytotoxic potential against two human prostatic adenocarcinomas (PC-3 and LNCaP), compared to a non-cancerous human prostate stromal myofibroblast cell line (WPMY-1) during the last ten years. Nowadays, the rate of prostate cancer is still increasing, despite the existence of several existing cancer therapies. It is one of the main reasons to have a constant view on the different phytochemicals previously active, which can be lead compounds and used in the development of new drugs that may fight against prostate cancer. We, therefore, noticed that the Clusiaceae family and *Garcinia* genus are rich source of xanthones and derivatives. The structure–activity relationship proposed in this work showed that the caged and neo-caged xanthones are a subclass with important activity against prostate cancer. Among the 51 bioactive xanthones reported in this work, garcibractatin A, gaudichaudione H, and cantleyanone A, which are all caged and neo-caged-prenylated xanthones, were the most potent against one prostate cancer cell line (PC-3) and one normal cancer cell line (WPMY-1). More in-depth analyses, such as in vivo studies using appropriate animal models, safety evaluations of non-cancerous cells, the mechanism of action as well as the hemi-synthesis of promising compounds by researchers in collaboration with some pharmaceutical companies, could be an interesting topic for the advancement of research to develop novel pharmaceutics against human prostatic cancer. Some impressive natural xanthone analogues, like psorospermin, an ingredient of the African plant *Psorospermum febrifugum*, show excellent anticancer activity against human and murine cancer cell lines. Because of the superb NCI 60 panel screening test results, psorospermin advanced to clinical trials, but further development for the commercial market suffered from limited resources.

## Figures and Tables

**Table 1:** Reported bioactive xanthones (**1**–**51**) against prostatic adenocarcinoma over the last ten years.

Characteristics of Included Studies						
Classes of Compounds	Xanthones	Plant Source (Family)	Year	Country	Type of Cancer Cells Lines	References
Simple oxygenated xanthones	Medicaxanthone (**1**)	*Citrus medica* (Rutaceae)	2015	Cameroon	Human PC (PC-3)	[24]
	Coxanthone B (**2**)	*Codonopsis ovata* (Campanulaceae)	2016	India	Human PC (PC-3)	[25]
Prenylated xanthones	Oliganthin H (**3**)	*Garcinia oligantha* (Clusiaceae)	2016	China	Human PC (PC-3)	[26]
	Oliganthin I (**4**)	*Garcinia oligantha* (Clusiaceae)	2016	China	Human PC (PC-3)	[26]
Caged prenylated-xanthones	Oliganthone B (**5**)	*Garcinia oligantha* (Clusiaceae)	2016	China	Human PC (PC-3)	[26]
	Garcibractatin A (**6**)	*Garcinia bracteate* (Clusiaceae)	2018	China	- Human PC (PC-3) - Normal human cell line (WPMY-1)	[28]
Prenylated xanthones	Bracteaxanthone VII (**7**)	*Garcinia bracteate* (Clusiaceae)	2018	China	- Human PC (PC-3) - Normal human cell line (WPMY-1)	[29]
	Bracteaxanthone VIII (**8**)	*Garcinia bracteate* (Clusiaceae)	2018	China	Human PC (PC-3)	[29]
	Paucinervin L (**9**)	*Garcinia paucinervis* (Clusiaceae)	2018	China	Human PC (PC-3)	[31]
Pyranoxanthones	(+) Paucinervin N (**10**)	*Garcinia paucinervis* (Clusiaceae)	2018	China	Human PC (PC-3)	[31]
	(−) Paucinervin N (**11**)	*Garcinia paucinervis* (Clusiaceae)	2018	China	Human PC (PC-3)	[31]
Prenylated xanthones	Paucinervin O (**12**)	*Garcinia paucinervis* (Clusiaceae)	2018	China	Human PC (PC-3)	[31]
	Paucinervin P (**13**)	*Garcinia paucinervis* (Clusiaceae)	2018	China	Human PC (PC-3)	[31]
Simple oxygenated xanthones	Lichenxanthone (**14**)	*Citrus medica* (Rutaceae)	2015	Cameroon	Human PC (PC-3)	[24]
	Swertiperenine (**15**)	*Codonopsis ovata* (Campanulaceae)	2016	India	Human PC (PC-3)	[25,27]
	1,7,8-Trihydroxy-3-methoxy-xanthone (**16**)	*Codonopsis ovata* (Campanulaceae)	2016	India	Human PC (PC-3)	[25]
Caged prenylated-xanthones	Gaudichaudione H (**17**)	*Garcinia oligantha* (Clusiaceae)	2016	China	Human PC (PC-3)	[26]
	Cantleyanone A (**18**)	*Garcinia oligantha* (Clusiaceae)	2016	China	Human PC (PC-3)	[26]
	Cochinchinoxanthone (**19**)	*Garcinia bracteate* (Clusiaceae)	2018	China	Human PC (PC-3)	[29]
	Bractatin (**20**)	*Garcinia bracteate* (Clusiaceae)	2018	China	Human PC (PC-3)	[29]
Caged prenylated-xanthones	1-*O*-methylbractatin (**21**)	*Garcinia bracteate* (Clusiaceae)	2018	China	- Normal human cell line (WPMY-1)	[29]
	Isobractatin (**22**)	*Garcinia bracteate* (Clusiaceae)	2018	China	Human PC (PC-3)	[29]
	1-*O*-methylisobractatin (**23**)	*Garcinia bracteate* (Clusiaceae)	2018	China	Human PC (PC-3)	[28]
	Epiisobractatin (**24**)	*Garcinia bracteate* (Clusiaceae)	2018	China	Human PC (PC-3)	[28]
	Forbesione (**25**)	*Garcinia bracteate* (Clusiaceae)	2018	China	Human PC (PC-3)	[28]
	Isoforbesione (**26**)	*Garcinia bracteate* (Clusiaceae)	2018	China	Human PC (PC-3)	[28]
Neo-caged prenylated-xanthones	Neobractatin (**27**)	*Garcinia bracteate* (Clusiaceae)	2018	China	Human PC (PC-3)	[28]
	3-*O*-methyl-neobractatin (**28**)	*Garcinia bracteate* (Clusiaceae)	2018	China	Human PC (PC-3)	[28]
Prenylated xanthones	Gartanin (**29**)	*Garcinia bracteate* (Clusiaceae)	2018	China	- Human PC (PC-3) - Normal human cell line (WPMY-1)	[28]
	3-Hydroxyblanco-xanthone (**30**)	*Garcinia bracteate* (Clusiaceae)	2018	China	- Human PC (PC-3) - Normal human cell line (WPMY-1)	[28]
	Xanthone V1 (**31**)	*Garcinia bracteate* (Clusiaceae)	2018	China	- Human PC (PC-3) - Normal human cell line (WPMY-1)	[28]
	Gerontoxanthone I (**32**)	*Garcinia bracteate* (Clusiaceae)	2018	China	- Human PC (PC-3) - Normal human cell line (WPMY-1)	[28]
	Xanthone V1a (**33**)	*Garcinia bracteate* (Clusiaceae)	2018	China	- Human PC (PC-3) - Normal human cell line (WPMY-1)	[28]
Simple oxygenated xanthones	1,5,6-trihydroxyxanthone (**34**)	*Mesua ferrea* (Calophyllaceae)	2019	Thailand	Human PC (PC-3)	[30]
Simple oxygenated xanthones	1,5-dihydroxy-6-methoxyxanthone (**35**)	*Mesua ferrea* (Calophyllaceae)	2019	Thailand	Human PC (PC-3)	[30]
	5-hydroxy-1-methoxyxanthone (**36**)	*Mesua ferrea* (Calophyllaceae)	2019	Thailand	Human PC (PC-3)	[30]
	5-hydroxy-1,3-dimethoxyxanthone (**37**)	*Mesua ferrea* (Calophyllaceae)	2019	Thailand	Human PC (PC-3)	[30]
Prenylated xanthones	Dulcisxanthone B (**38**)	*Cratoxylum cochinchinense* Blume (Clusiaceae)	2019	China	Human PC (PC-3)	[31]
	Cudratricusxanthone E (**39**)	*Cratoxylum cochinchinense* Blume (Clusiaceae)	2019	China	Human PC (PC-3)	[31]
	γ-Mangostin (**40**)	*Cratoxylum cochinchinense* Blume (Clusiaceae)	2019	China	Human PC (PC-3)	[31]
Prenylated xanthones	1,3,7-trihydroxy-2,4-Diisoprenylxanthone (**41**)	*Cratoxylum cochinchinense* Blume (Clusiaceae)	2019	China	Human PC (PC-3)	[31]
	Cochinchinone A (**42**)	*Cratoxylum cochinchinense* Blume (Clusiaceae)	2019	China	Human PC (PC-3)	[31]
	Cochinchinone B (**43**)	*Cratoxylum cochinchinense* Blume (Clusiaceae)	2019	China	Human PC (PC-3)	[31]
	Pruniflorone Q (**44**)	*Cratoxylum cochinchinense* Blume (Clusiaceae)	2019	China	Human PC (PC-3)	[31]
Pyranoxanthone	Pruniflorone N (**45**)	*Cratoxylum cochinchinense* Blume (Clusiaceae)	2019	China	Human PC (PC-3)	[31]
Prenylated xanthones	Xanthone V1 (**46**)	*Cratoxylum cochinchinense* Blume (Clusiaceae)	2019	China	Human PC (PC-3)	[31]
Prenylated xanthones	Parvifolixanthone A (**47**)	*Garcinia paucinervis* (Clusiaceae)	2018	China	Human PC (PC-3)	[32]
	2-prenyl-1,3,5,6-tetrahydroxylxanthone (**48**)	*Garcinia paucinervis* (Clusiaceae)	2018	China	Human PC (PC-3)	[32]
	7-prenyljacareubin (**49**)	*Garcinia paucinervis* (Clusiaceae)	2018	China	Human PC (PC-3)	[32]
	Paucinervin I (**50**)	*Garcinia paucinervis* (Clusiaceae)	2018	China	Human PC (PC-3)	[32]
	Subelliptenones F (**51**)	*Garcinia subelliptica* (Clusiaceae)	2014	Japan	Human PC (LNCaP)	[33]

WPMY-1: Normal Human Prostatic stromal Myofibroblast cell line; PC-3: Human Prostate Cancer; LNCaP: Lymph Node Carcinoma of the Prostate.

**Table 2:** Reported xanthones with their mechanism of action on the different cell lines.

Compounds	Mechanism of Action	Cell Lines	Targets	References
Isobractatin (**22**)	Enhancement of the cell apoptosis, and arrested cell cycle in the G0/G1 phase.	Human prostate cancer (PC-3)	↓ Cyclin D1 and E, ↑ CDK, ↑ P21, ↑ Bax, ↑ Caspase 3 and 9, ↓ Bcl-2.	[69]
γ-Mangostin (**40**)	Promote cell cycle arrest and apoptosis.	Human prostate carcinoma epithelial cell line (22Rv1)	↓ CDK2/CyclinE1	[59]

↓: Downregulation and ↑: Upregulation.

**Table 3:** Database of bioactive reported xanthones.

Classes of Compounds	Name of Compounds	Molecular Formula	Molecular Mass (Cal.)
Oxygenated-xanthones	Medicaxanthone (**1**)	C_47_H_66_O_8_	758.4758
	Coxanthone B (**2**)	C_19_H_20_O_6_	344.1260
	Lichenxanthone (**14**)	C_16_H_14_O_5_	286.0841
	Swertiperenine (**15**)	C_15_H_12_O_6_	288.0634
	1,7,8-Trihydroxy-3-methoxy-xanthone (**16**)	C_14_H_10_O_6_	275.0477
	1,5,6-trihydroxyxanthone (**34**)	C_29_H_34_O_6_	244.0372
	1,5-dihydroxy-6-methoxyxanthone (**35**)	C_14_H_10_O_5_	258.0528
	5-hydroxy-1-methoxyxanthone (**36**)	C_14_H_10_O_4_	242.0579
	5-hydroxy-1,3-dimethoxyxanthone (**37**)	C_15_H_12_O_5_	272.0685
Prenylated-xanthones	Oliganthin H (**3**)	C_33_H_38_O_7_	546.2618
	Oliganthin I (**4**)	C_28_H_30_O_7_	478.1992
	Bracteaxanthone VII (**7**)	C_27_H_26_O_6_	410.1729
	Bracteaxanthone VIII (**8**)	C_23_H_24_O_6_	396.1573
	Paucinervin L (**9**)	C_29_H_32_O_7_	492.2148
	Paucinervin O (**12**)	C_23_H_22_O_6_	394.1416
	Paucinervin P (**13**)	C_25_H_24_O_6_	420.1573
	Gartanin (**29**)	C_23_H_24_O_6_	396.1573
	3-Hydroxyblanco-xanthone (**30**)	C_23_H_22_O_6_	394.1416
	Xanthone V1 (**31**)	C_23_H_22_O_6_	394.4230
	Gerontoxanthone I (**32**)	C_23_H_24_O_6_	396.1576
	Xanthone V1a (**33**)	C_23_H_24_O_6_	396.1573
	Dulcisxanthone B (**38**)	C_24_H_26_O_6_	410.1729
Prenylated-xanthones	Cudratricusxanthone E (**39**)	C_23_H_24_O_6_	396.1573
	γ-Mangostin (**40**)	C_23_H_24_O_6_	396.1573
	1,3,7-trihydroxy-2,4-diisoprenylxanthone (**41**)	C_23_H_24_O_5_	380.1624
	Cochinchinone A (**42**)	C_28_H_32_O_5_	448.2250
	Cochinchinone B (**43**)	C_28_H_32_O_6_	465.2199
	Pruniflorone Q (**44**)	C_28_H_32_O_6_	464.2199
	Xanthone V1 (**46**)	C_23_H_22_O_6_	394.1416
	Parvifolixanthone A (**47**)	C_28_H_32_O_6_	464.2199
	2-prenyl-1,3,5,6-tetrahydroxylxanthone (**48**)	C_18_H_16_O_6_	328.0947
	7-prenyljacareubin (**49**)	C_23_H_22_O_6_	394.1416
	Paucinervin I (**50**)	C_23_H_22_O_6_	394.1416
	Subelliptenones F (**51**)	C_18_H_16_O_6_	328.0947
Caged-prenylated-xanthones	Oliganthone B (**5**)	C_28_H_32_O_7_	480.2148
	Garcibractatin A (**6**)	C_30_H_36_O_6_	492.2512
	Gaudichaudione H (**17**)	C_29_H_34_O_7_	494.2305
	Cantleyanone A (**18**)	C_34_H_42_O_7_	562.2931
	Cochinchinoxanthone (**19**)	C_23_H_24_O_6_	396.1573
	Bractatin (**20**)	C_28_H_32_O_6_	464.2199
	1-*O*-methylbractatin (**21**)	C_29_H_34_O_6_	478.2355
	Isobractatin (**22**)	C_28_H_32_O_6_	464.2199
	1-*O*-methylisobractatin (**23**)	C_29_H_34_O_6_	478.2355
	Epiisobractatin (**24**)	C_28_H_32_O_6_	464.2199
	Forbesione (**25**)	C_28_H_32_O_6_	464.2199
	Isoforbesione (**26**)	C_28_H_32_O_6_	464.2199
Neo-caged-prenylated-xanthones	Neobractatin (**27**)	C_28_H_32_O_6_	464.2199
	3-*O*-methyl-neobractatin (**28**)	C_29_H_34_O_6_	478.2355
Pyrano-xanthone	(+) Paucinervin N (**10**)	C_23_H_22_O_8_	426.1215
	(–) Paucinervin N (**11**)	C_23_H_22_O_6_	394.1416
	Pruniflorone N (**45**)	C_18_H_16_O_6_	328.0947

## Data Availability

Not applicable.
